# The impact of early life maternal deprivation on the perineuronal nets in the prefrontal cortex and hippocampus of young adult rats

**DOI:** 10.3389/fcell.2022.982663

**Published:** 2022-11-28

**Authors:** Ana Jakovljevic, Gorana Agatonovic, Dubravka Aleksic, Milan Aksic, Gebhard Reiss, Eckart Förster, Antonios Stamatakis, Igor Jakovcevski, Joko Poleksic

**Affiliations:** ^1^ Center for Laser Microscopy, Institute for Physiology and Biochemistry “Jean Giaja”, Faculty of Biology, University of Belgrade, Belgrade, Serbia; ^2^ School of Medicine, Institute of Anatomy “Niko Miljanic”, University of Belgrade, Belgrade, Serbia; ^3^ Institut für Anatomie und Klinische Morphologie, Universität Witten/Herdecke, Witten, Germany; ^4^ Department of Neuroanatomy and Molecular Brain Research, Institute of Anatomy, Ruhr-Universität Bochum, Bochum, Germany; ^5^ Biology-Biochemistry Lab, School of Health Sciences, Faculty of Nursing, National and Kapodistrian University of Athens, Athens, Greece

**Keywords:** perineuronal nets, interneurons, maternal deprivation, early life stress, prefrontal cortex, hippocampus

## Abstract

Early life stress negatively impacts brain development and affects structure and function of parvalbumin immunopositive (PV+) inhibitory neurons. Main regulators of PV+ interneurons activity and plasticity are perineuronal nets (PNNs), an extracellular matrix formation that enwraps PV+ interneurons mainly in the neocortex and hippocampus. To experimentally address the impact of early life stress on the PNNs and PV+ interneurons in the medial prefrontal cortex and dorsal hippocampus in rats, we employed a 24 h maternal deprivation protocol. We show that maternal deprivation in the medial prefrontal cortex of adult rats caused a decrease in density of overall PNNs and PNNs that enwrap PV+ interneurons in the rostral cingulate cortex. Furthermore, a staining intensity decrease of overall PNNs and PNN+/PV+ cells was found in the prelimbic cortex. Finally, a decrease in both intensity and density of overall PNNs and PNNs surrounding PV+ cells was observed in the infralimbic cortex, together with increase in the intensity of VGAT inhibitory puncta. Surprisingly, maternal deprivation did not cause any changes in the density of PV+ interneurons in the mPFC, neither had it affected PNNs and PV+ interneurons in the hippocampus. Taken together, our findings indicate that PNNs, specifically the ones enwrapping PV+ interneurons in the medial prefrontal cortex, are affected by early life stress.

## Introduction

Maternal deprivation (MD) is one of the most commonly used animal models of early life stress. As thoroughly reviewed by Marco et al., 2015 stress in early age in a form of 24 h separation on postnatal day (PND) nine presents a reliable paradigm for examining psychopathologies with developmental origins, such as schizophrenia and depression. Indeed, the prevalence of adversities during childhood such as neglect, emotional, sexual and physical abuse is remarkably increased in adult depressive and schizophrenic patients (Bentall et al., 2012; Mandelli et al., 2015; Infurna et al., 2016). Furthermore, early life stress has been associated with morphological changes in the human brain. Thus, gray matter volume reductions have been found in the medial prefrontal cortex (mPFC), hippocampus and amygdala of subjects with self-reported history of childhood abuse (Edmiston, 2011; Gorka et al., 2014; Underwood et al., 2019). It has been suggested that early life adversity interferes with the normal trajectory of brain development and increases vulnerability to stress. As a result, another stressful event that occurs later in life may trigger the onset of a mental disorder (Maynard et al., 2001; McEwen and Morrison, 2013).

Previously, it has been found that 24 h MD reduces total neuron density in the PFC of young adult male rats (Aksić et al., 2013). Furthermore, GABAergic cells are also affected in the hippocampus and PFC of MD rats in a subregion- and subpopulation-specific manner (Aksic et al., 2021; Poleksic et al., 2021). Interestingly, morphological changes are accompanied by microglial activation, although a specific nature of glial response seems to differ between various models of early life stress (Ferle et al., 2020; Wang et al., 2020; Poleksic et al., 2021) Apart from changes in neuronal and glial densities, MD leads to long–term functional deficits such as impairment in cognitive flexibility (Baudin et al., 2012), recognition memory (Marco et al., 2013), and social behavior (Holland et al., 2014).

Perineuronal nets (PNNs) are condensed extracellular matrix mesh-like structures that enwrap mainly parvalbumin immunopositive (PV+) cell bodies and proximal dendrites in the cortex (Härtig et al., 1992) and hippocampus (Celio and Chiquet-Ehrismann, 1993). They are composed of interconnected molecules of hyaluronic acid, chondroitin sulfate proteoglycans, linker proteins and glycoproteins, secreted both by neurons (Härtig et al., 1992) and glial cells (Brückner et al., 1993). PV expression depends on the presence or absence of PNNs, thus in the hippocampus intensly stained PV+ interneurons tend to be surrounded by PNNs, while weakly stained PV+ interneurons lack PNNs (Yamada et al., 2015). Moreover, PNNs presence around PV+ neurons is associated with a higher density of perisomatic inhibitory and excitatory synapses, larger somata (Enwright et al., 2016), and longer axonal initial segments in the mPFC (Carceller et al., 2020). Strikingly, maturation of PNNs, reflected through an increase in staining intensity, coincides with the closure of the critical period in brain development (Pizzorusso, 2002). On the ultrastructural level, as animals become older and PNNs mature, holes in the PNNs mesh become smaller, tightening the grip on the synapses that perforate PNNs and project onto the neuron (Sigal et al., 2019). Through control of synapse formation and its stabilization on the PV+ interneurons, PNNs regulate PV+ interneuron excitability as well as GABA release (Wang and Fawcett, 2012; Yamada et al., 2015). Proper expression of both PV and formation of PNNs is crucial for normal brain development (Mix et al., 2015).

Maternal deprivation (MD) can influence normal PNNs and PV development, causing alteration in number and intensity (Gildawie et al., 2020). Affected regions are included in corticolimbic circuits (Riga et al., 2017; Page et al., 2018) and changes can be sex-specific, which is shown in Soares et al., 2020 where maternal separation during neonatal period for 4 h/day from PND2-20 caused a decrease in PV+ cell density only in adolescent male but not female mPFC. Furthermore, following 4 h/day MD between PND2-20, adult females had reduced PNN density mainly in infralimbic (IL) but not in prelimbic (PrL) cortex (Gildawie et al., 2020). Besides the mPFC, reduction of PV+ staining intensity after MD was found in the amygdala (Soares et al., 2020) and the ventral hippocampus (Murthy et al., 2019), accompaned by a decrease in PV + cell density as well. Dorsal hippocampus receives monosynaptic projection from mPFC (Rajasethupathy et al., 2016). Presumably, this pathway is critical for cognitive functions such as working memory (Izaki et al., 2008) and spatial learning, while ventral hippocampus—PFC interactions are implicated in emotional behaviors (Bannerman et al., 2004). In addition, PNNs allow normal firing of PV+ interneurons (Wingert and Sorg, 2021), thus emphasizing their important role in cognitive regulation (Ferguson and Gao, 2018), which seems to be affected by early life stress (Janetsian-Fritz et al., 2018).

Balance between excitatory and inhibitory neurotransmission in the mPFC that underlies healthy cognitive and emotional functioning is established in early postnatal and adolescent development. Main regulator of that balance is GABA-ergic system of interneurons, mainly PV expressing, enwrapped by PNNs (Page and Coutellier, 2018). It has been shown that various stress protocols, such as early life stress (Castillo-Gómez et al., 2017) and peripubertal stress (Page and Coutellier, 2018) can strongly influence sensitive excitation/inhibition balance in the PFC and amygdala. Abnormal inhibition is one of the important findings in schizophrenic patients (Chen et al., 2014).

This study investigated long term structural changes in the mPFC and dorsal hippocampus after maternal deprivation on PND9 for 24 h, a widely used paradigm for the investigation of the influence of early life stress on brain development (Ellenbroek et al., 2004). In order to adress such changes, we examined, density and intensity of PNNs, around PV+ and non-PV+ (PV−) cells PV+ interneuron density, as well as volume of the three mPFC (rostral cingulate-roCg1, prelimbic-PrL and infralimbic-IL cortices) and the three hippocampal (CA1, CA3, dentate gyrus) subregions using immunohistochemistry and cresyl violet staining, respectively. Also, for the first time the number and intensity of VGAT inhibitory terminals projected on PNN+/PV+ cells was examined in the mPFC in this particular MD model.

## Material and methods

All efforts were made to minimize animal suffering and to reduce the number of animals used in the study. All experiments were carried out according to the NIH Guide for Care and Use of Laboratory Animals and were approved by the Ethics Committee of the University of Belgrade.

### Animals

For the purpose of this experiment, we used Wistar albino rats kept in standard plexiglass cages (26 × 42 × 15 cm) with wire lid and sawdust, in a temperature (23 ± 1°C) and humidity (40%–70%) controlled facility. The animals were maintained in a standard 12 h light/dark cycle (lights on from 07:00 to 19:00), with tap water and food available *ad libitum.* Animals were mated (1 male x 2 females) and after approximately 14 days if the pregnancy was confirmed, the dam was individually housed and the day of delivery (PND0) strictly controlled. Prior to delivery, each litter was randomly assigned to the control or MD group. No culling procedure was performed. All dams and litters were left undisturbed except for the routine cleaning of the cages and the MD procedure. On PND22, animals were weaned and housed in same sex, same group (control, MD) of 3–4 animals per cage. Only male rats were used in the experiment, and number of animals per group (control vs. MD) was 5. To compensate for any litter effects, rats derived from three different litters were used for morphological analysis.

### Neonatal stress

**FIGURE 1:**
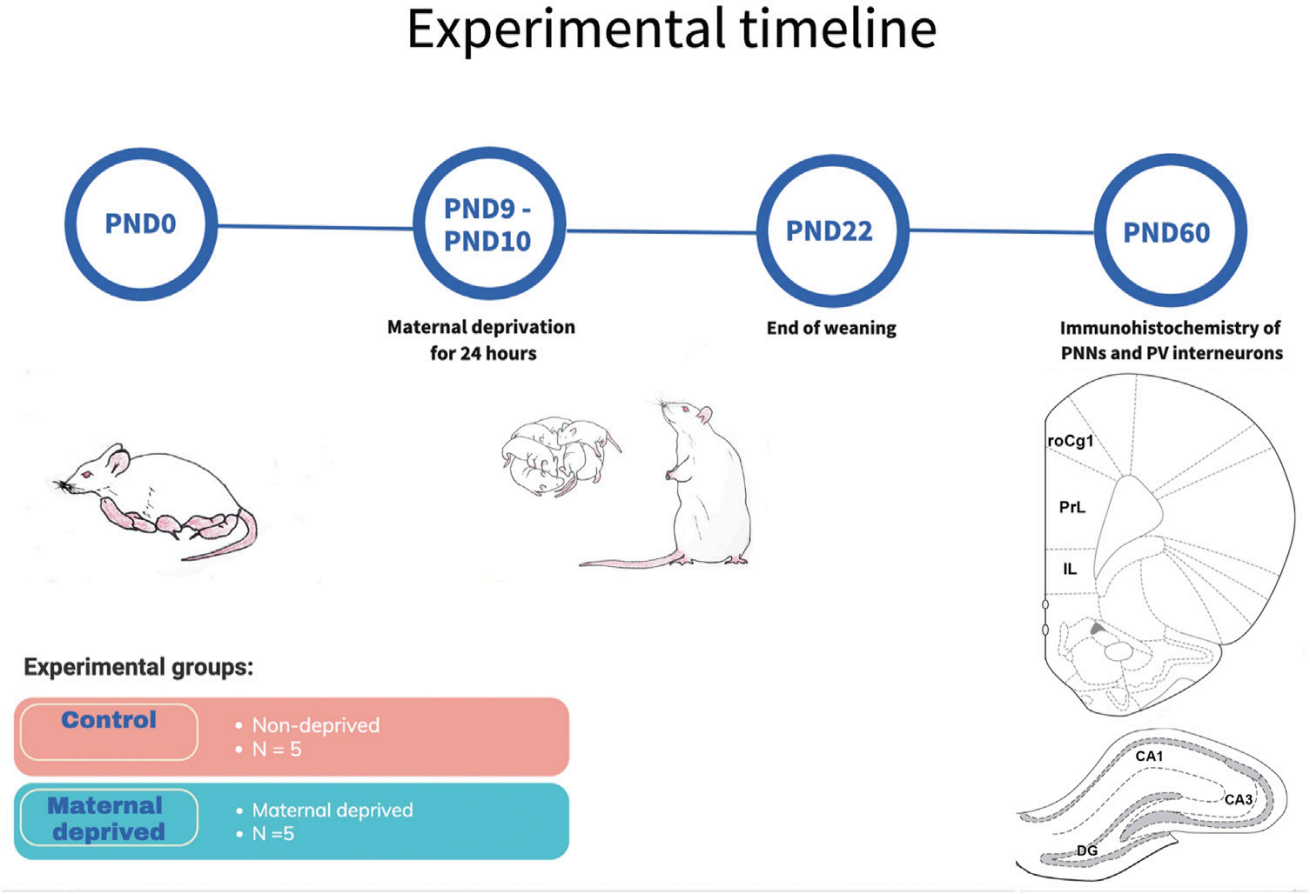
Schematic representation of experimental timeline. On PND9, dams have been removed from pups for 24 h, and brought back until weaning on PND22. Brains from young adult males from three differend litters were used for immunohistochemical analysis of PNNs and PV + interneurons in three regions of the medial prefrontal cortex and three regions of the dorsal hippocampus. Hippocampal subfields CA1 (CA1) and CA3 (CA3), rostral part of the area one of the cingulate cortex (roCg1), dentate gyrus (DG), infralimbic cortex (IL), maternally deprived rats (MD), medial prefrontal cortex (mPFC), prelimbic cortex (PrL), postnatal day (PND).

As previously described in detail (Ellenbroek et al., 2004; Marco et al., 2015), the MD procedure was performed on PND9 (Figure 1). In brief, at 10:00 a.m. dams were removed from the cage and placed in a separate cage, in a separate room. Pups were left undisturbed for 24 h when dams were returned to their corresponding litters in the home cage (PND10 at 10:00 a.m.). In control litters, pups were handled during a brief separation (3 min) on both PND 9 and 10.

### Tissue processing for histology

For morphological analysis, on PND60, young adult rats (*n* = 5 for both groups) were anesthetized with ketamine xylazine solution (100 mg/kg body weight; 33 mg/kg body weight) and transcardially perfused with 150–200 ml of 0.9% saline followed by 220–250 ml of fixative (4% paraformaldehyde in 0.1 M phosphate buffer pH 7.4). After decapitation, brains were extracted, post-fixed for 24 h at +4°C, cryoprotected by infiltration with sucrose for 48 h at +4°C (30% sucrose in 0.1 M phosphate buffer) and stored at −80°C until sectioning. Serial coronal sections of 25 μm in thickness were cut on a freezing cryostat (Leica Instruments, Nussloch, Germany) at −25°C, collected on SuperFrost Plus glass slides (Menzel, Braunschweig, Germany) in a spaced serial sequence (5–6 sections 250 μm apart were present on each slide) and stored at -20°C until use.

### Immunohistochemistry

For PV and WFA immunofluorescence, after thawing, sections were rehydrated in phosphate buffer saline (PBS) (3 × 10 min). Non-specific binding was blocked with 10% normal donkey serum (NDS) and 1% bovine serum albumin (BSA) in 0.3% Triton X-100 PBS for 1 h at room temperature. Sections were incubated with lectin from Wisteria floribunda agglutinin (WFA, 1:100; L1516, MilliporeSigma, United States) and anti-PV (mouse monoclonal, 1:1,000; MAB1572, MilliporeSigma, United States) antibody diluted in 0.3% Triton X-100 PBS with 2% NDS and 1% BSA overnight at 4°C. Following incubation, sections were washed in PBS (3 × 10 min) and incubated for 2 h at room temperature in the dark with Alexa 488-conjugated streptavidin (1:200; S11223, Thermofisher, United States) and Alexa 555-conjugated donkey anti-mouse (1:200; A31570, Invitrogen, United States) secondary antibody diluted in PBS containing 2% BSA. Following five subsequent washes in PBS, nuclear staining was performed using diamidino-2-phenylindole (DAPI, 1:4,000; 18,860.01, Serva, Germany) for 10 min in the dark at room temperature. Afterwards, sections were again thoroughly washed in PBS, coverslipped using Mowiol mounting medium and allowed to dry-out overnight before analysis. For WFA, VGAT, and PV triple immunofluorescence, after thawing, sections were rehydrated in PBS (30 min) and antigen retrieval procedure was performed in citrate buffer pH6 for 10 min. Sections were rinsed in PBS (2 × 5 min) and non-specific binding was blocked with 20% NDS in PBS for 1 h at room temperature. Sections were incubated with lectin from WFA (1:100), anti-PV (1:1,000) and anti-VGAT (rabbit polyclonal, 1:200,131 003, Synaptic Systems) antibody in 10% NDS and 0.3% Triton X-100 in PBS for 30 h at 4°C. After incubation, sections were washed in PBS (3 × 15 min) and incubated in dark for 2 h at room temperature with Alexa 488-conjugated streptavidin (1:200), Alexa-555 conjugated donkey anti-rabbit (1:200, A31572, Invitrogen) and Alexa-647 conjugated donkey anti-mouse (1:200, A31571, Invitrogen) antibody. Following incubation, sections were rinsed in PBS (4 × 15 min), coverslipped using Mowiol mounting medium, and dry-out overnight. To confirm that immunohistochemistry stainings for all markers were specific, negative controls have been performed by omission of primary antibodies, and by replacement of primary antibodies with the equal concentration of the normal serum from the same species, which resulted in the absence of staining.

### Image acquisition and cell counting

WFA+ PNNs and PV+ cells were estimated in mPFC and dorsal hippocampus. Expression of VGAT puncta on the WFA+ PNNs around PV+ cells were estimated only in the mPFC. Each brain area was analyzed on 5–12 systematic randomly selected sections (depending on the brain area analyzed) within anatomical borders, according to the anatomical atlas of Paxinos and Watson (Table 1) (Paxinos and Charles Watson, 2006). 2D image acquisition was performed on confocal laser-scanning microscope (LSM 510, Carl Zeiss, GmbH, Jena, Germany), equipped with Ar (488 nm) and HeNe (543 nm, 633 nm), lasers using 40X (Plain Apochromant, NA = 1.3) and 63X (DIC, NA = 1.4) oil immersion objective. Imaging settings were identical for every experimental group allowing comparisons of pixel intensities between groups. Images were analyzed in FIJI, an open source image processing package based on ImageJ (ImageJ v.1.46R, NIH, United States). In each section, the density of PV+ interneurons, density and intensity of WFA+ PNNs (PNN+), WFA+ PNNs colocalized with PV (PNN+/PV+) and WFA+ PNNs not colocalizing with PV (PNN+/PV−) were counted in two randomly selected non-overlapping cortical (one in the superficial, one in the deep layer) or hippocampal subfields, optical fields of 106,113 μm^2^. Cells were counted manually by one observer blind to the experimental conditions.

**TABLE 1:** Anatomical borders of analyzed brain areas.

Brain area	Abbreviation	Anatomical borders in mm (relative to bregma)	Number of sections analyzed
*mPFC*			
rostral part of the cingulate cortex	roCg	4.20 to 2.52	6–8
prelimbic cortex	PrL	5.16 to 2.52	8–12
infralimbic cortex	IL	3.72 to 2.52	3–5
*Hippocampus*			
CA1 of the hippocampus	CA1
CA3 of the hippocampus	CA3	−1.92 to −3.72	4–9
dentate gyrus	DG		

Intensity of PNNs was analyzed firstly by removing background staining using rolling ball radius function (Murthy et al., 2019), followed by creation of ROI for each PNN separately, measuring mean of pixel intensity in selected ROI, with pixel values in range 0–255.

Estimation of perisomatic VGAT puncta on the PNN+/PV+ cells in the mPFC was performed by imaging 1 cell at the level of the largest cell body cross-sectional area with ×63 objective and digital zoom of 1.5. Counting individually discernible perisomatic VGAT puncta and measuring their pixel intensity was performed using ImageJ (Papastefanaki et al., 2015).

### Volume estimation

Cresyl violet staining was used for quantification of mPFC and the hippocampus volume, divided into subsections. In short, brain sections were incubated into xylene (5 min), 95% ethanol (3 min), 70% ethanol (3 min) and rinsed in distilled water. Cresyl violet dye (10,510-54-0, Sigma Aldrich) was applied for 10 min at 60°C, rinsed in distilled water and incubated in increasing gradient of ethanol concentration (70, 95, and 100%) for 3 min each, finalized with a xylene incubation for 5 min, prior to drying and applying mounting medium.

For estimation of the mPFC and the hippocampus volumes, micrographs were imaged using digital microscope camera Leica EC3 with magnification ×2. Volumetric analysis was performed by delineating the mPFC into the roCg1, PrL and IL, and the hippocampus into the CA1, CA3, and DG subregions in ImageJ and their volume was calculated using Cavalieri’s principle (Prakash et al., 1994).

### Statistical analyses

The density and intensity quantifications have been analyzed by the generalized linear model (GLM), and generalized estimating equation (GEE), with the treatment as predictor factor and the treatment (litter) as a build nested predictor factor. GLM and GEE were chosen as statistical tests since they take into consideration litter effects. Distribution of data and homogeneity of variances have been tested with Kolmogorov-Smirnof, Shapiro-Wilk and Leven’s tests, respectively. Grubb’s test for outliers has been performed, and we analyzed dataset without outliers for intensity quantification with Mann-Whitney as non-parametric test. The level of statistical significance was set at 0.001 (*p* = 0.05/54, the total number of statistical comparisons). The cut-off *p*-value was adjusted (lowered) in order to compensate for multiple statistical tests and thus minimize the chances of type-I errors. All tests were performed with the SPSS software (Release 22, SPSS, United States), and graphics were made in Interactive Dotplot (Weissgerber et al., 2017) and Numbers (Apple, United States).

## Results

The aim of this study was to examine long term effects of 24 h neonatal MD in young adult male rats (PND60, *n* = 5 for each group) on perineuronal nets and inhibitory synaptic terminals in the medial prefrontal cortex and hippocampus. Firstly, the density and staining intensity of PNNs around PV+ and PV− neurons in the mPFC and hippocampus have been investigated. Next, we examined number and intensity of VGAT inhibitory puncta around PNN+/PV+ cells in the mPFC. Finally, volume of mPFC and hippocampus in control and MD animals were measured. In order to examine subregion specific changes mPFC was divided into roCg1, PrL and IL, and the hippocampus was divided into DG, CA3, and CA1 subfields. Total number of evaluated PV + cells, PNNs and PNNs with VGAT puncta for the mPFC and hippocampus are presented in Supplementary Tables S2, S3, respectively.

### Maternal deprivation altered the density of PNNs in the mPFC without affecting the hippocampus

**FIGURE 2:**
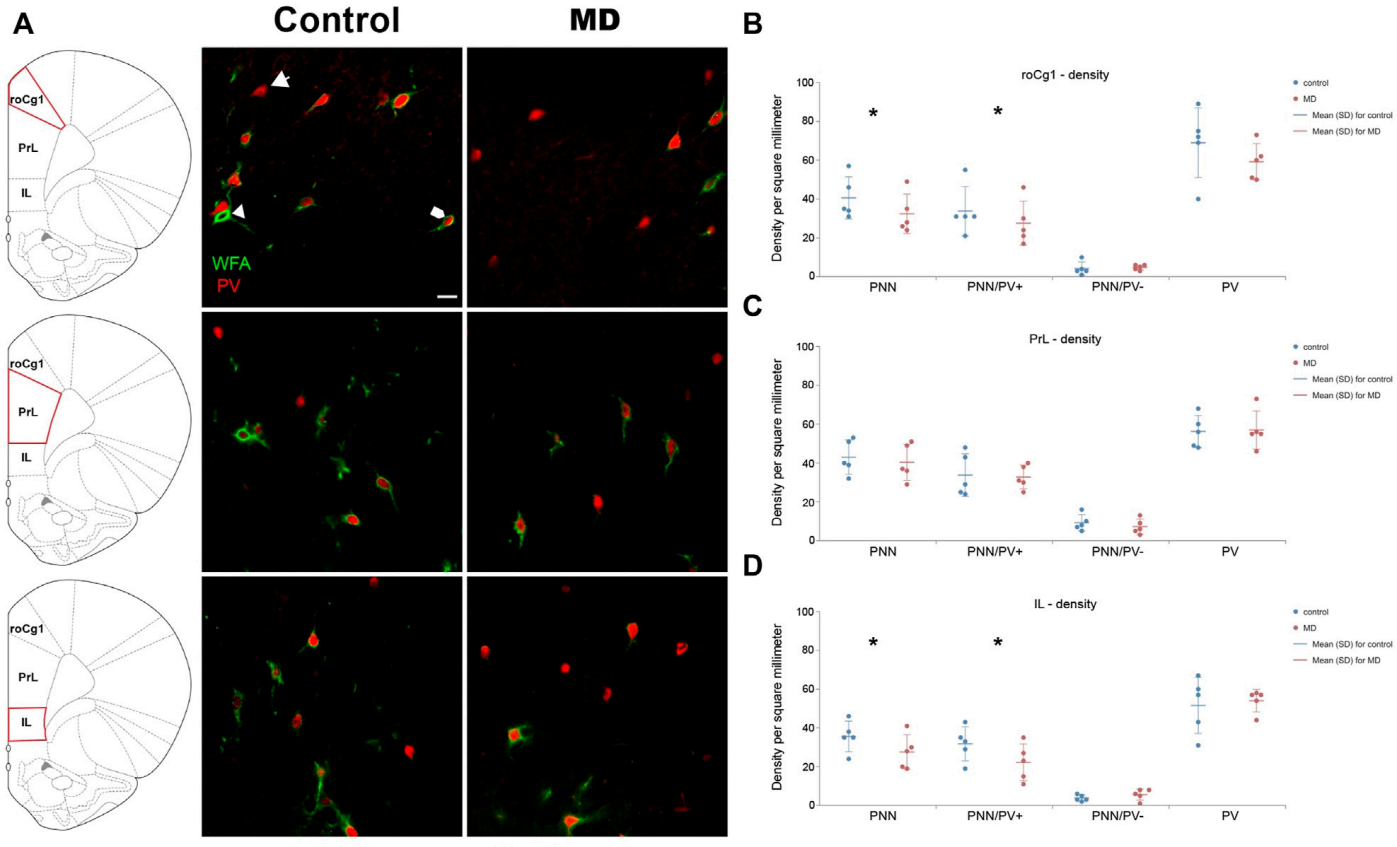
Effects of MD on WFA + perineuronal nets and PV + cell density in the mPFC on PND60. **(A)** Photomicrographs of double WFA/PV immunofluorescence showing PNNs surrounding PV + cells (pentagonal arrow), PNNs surrounding PV- cells (arrowhead) and PV + cells lacking PNNs (arrow) in the roCg1, PrL and IL. *Dot plot graphs* in **(B–D)** representing quantifications of results as means ± SD (horizontal bars) and observed values (dots) in the roCg1, PrL and IL, respectively. MD reduced density of overall PNNs and PNNs enwrapping PV + cells in the roCg1 **(B)** and IL **(D)**. Average number of evaluated PNNs per animal: roCg1 = 54, PrL = 89, IL = 24. *p < 0.001 Control vs. MD. Rostral part of area one of cingulate cortex (roCg1), infralimbic cortex (IL), maternally deprived rats (MD), prelimbic cortex (PrL). Scale bar in A = 25 μm.

**FIGURE 3:**
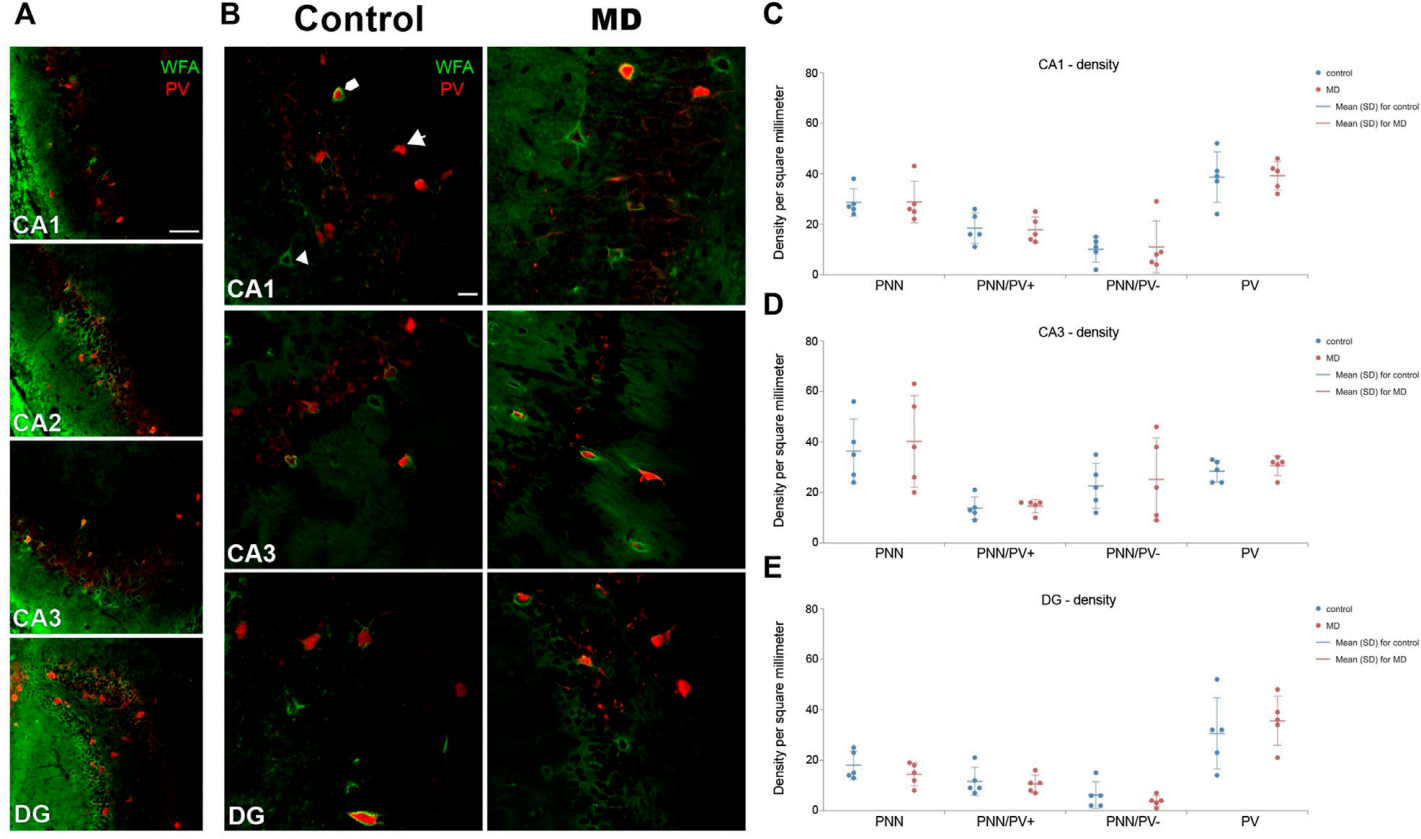
Effects of MD on WFA + perineuronal nets and PV + cell density in the hippocampus on PND60. **(A)** Low magnification photomicrographs of double WFA/PV + immunofluorescence showing CA1, CA2, CA3, and DG. Note the characteristic “mesh-like” appearance of PNNs in CA2. **(B)** High magnification photomicrographs of double WFA/PV + immunofluorescence showing PNNs surrounding PV + cells (pentagonal arrow), PNNs surrounding PV- cells (arrowhead) and PV + cells lacking PNNs (arrow) in CA1, CA3, and DG. *Dot plot graphs* in **(C–E)** representing quantification of results as means ± SD (horizontal bars) and observed values (dots) in the CA1, CA3 and DG, respectively. MD did not alter density of PNNs nor PV + cells in any of the examined areas. Average number of evaluated PNNs per animal: CA1 = 36, CA3 = 42, DG = 21. Hippocampal subfields CA1 (CA1), CA2 (CA2), and CA3 (CA3), dentate gyrus (DG), maternally deprived rats (MD). Scale bars in A and B = 100 and 25 μm, respectively.

We first examined the densities of perineuronal nets in the medial prefrontal cortex and hipopocampus. MD induced changes of the density of PNNs in the mPFC in an area-specific manner. Overall, PNN+ and PNN+/PV+ densities were significantlly decreased in the roCg1 (GLM, control mean ± SD = 40.55 ± 10.57/mm^2^ vs. MD mean ± SD = 32.47 ± 10.45/mm^2^, *n* = 5, *p* < 0.001 and control = 33.98 ± 12.68/mm^2^ vs. MD = 27.60 ± 11.44/mm^2^, *n* = 5, *p* < 0.001, respectively; Figures 2A,B) and IL (GLM, control = 35.57 ± 7.71/mm^2^ vs. MD = 27.62 ± 8.89/mm^2^, *n* = 5, *p* < 0.001 and control = 31.72 ± 8.81/mm^2^ vs. MD = 22.03 ± 9.46/mm^2^, *n* = 5, *p* < 0.001, respectively; Figures 2A,D) of young adult MD rats compared to controls, while no differences were found in the PrL (Figures 2A,C). The density of PNNs enwrapping PV− cells as well as the density of PV+ cells were not affected in any of the examined mPFC areas in MD rats. Additionally, we found no changes in PNN+, PNN+/PV+, and PNN+/PV− density in the CA1, CA3 or DG of MD rats compared to controls. Same as for the mPFC, MD did not induce changes in the hippocampal PV+ cell density (Figure 3). We conclude that maternal deprivation decreases densities of perineuronal nets around parvalbumin-expressing interneurons in the medial prefrontal cortex, without affecting the hippocampus.

### Maternal deprivation reduced PNN+ staining intensity in the mPFC, but not in the hippocampus

**FIGURE 4:**
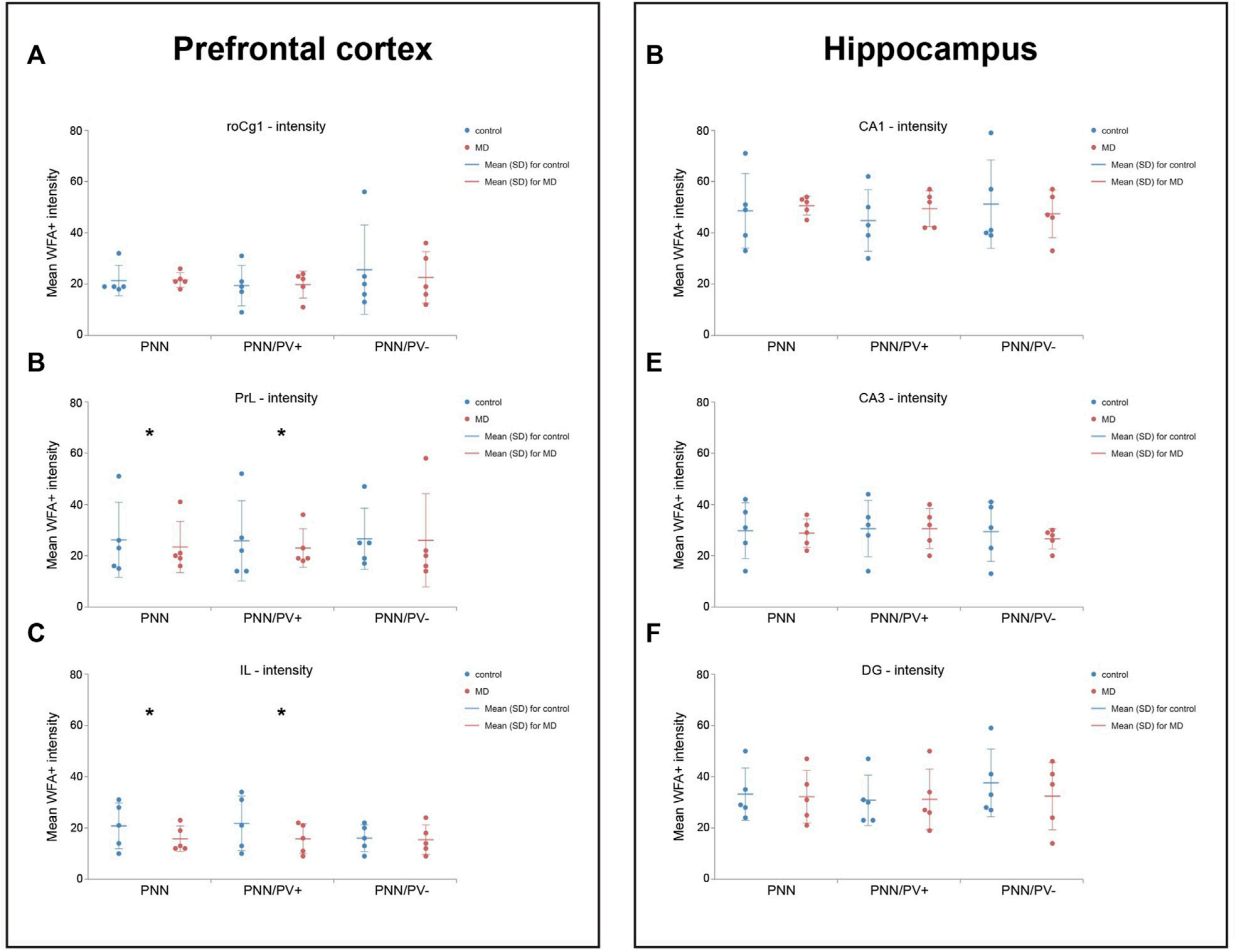
Effects of MD on WFA + perineuronal nets intensity in the mPFC and hippocampus. *Dot plot graphs* representing quantifications of the results as means ± SD (horizontal bars) and observed values (dots) in the respective mPFC **(A–C)** or hippocampal areas **(D–F)**. MD rats had lower intensity of overall PNNs and PNNs surrounding PV + cells in the PrL **(B)** and IL **(C)**. *p < 0.001 Control vs. MD. Hippocampal subfield CA1 (CA1) and CA3 (CA3), rostral part of area one of cingulate cortex (roCg1), dentate gyrus (DG), infralimbic cortex (IL), maternally deprived rats (MD), prelimbic cortex (PrL).

As the surface density of the PNNs was decreased, we asked whether the intensity of staining was changed at the single neuron level as well. Similar to the density changes, MD affected the intensity of PNNs in an area specific manner. Namely, in the PrL and IL, overall PNN + staining intensity (GLM, control = 26.30 ± 14.71 vs. MD = 23.45 ± 9.84, *n* = 5, *p* < 0.001 and control = 20.73 ± 9.03 vs. MD = 15.65 ± 4.96, *n* = 5, *p* < 0.001 respectively), as well as the intensity of PNNs surrounding PV + neurons (GLM, control = 25.70 ± 15.91 vs. MD = 22.73 ± 7.48, *n* = 5, *p* < 0.001 and control = 21.81 ± 10.77 vs. MD = 15.79 ± 5.81, *n* = 5, *p* < 0.001, respectively) were significantly reduced in young adult male MD rats when compared to controls, while no differences were observed in the roCg1 (Figures 4A–C). As in PrL region Grubb’s test confirmed outliers, and non-parametric Mann-Withney test indicated the difference was not statisticlly significant (*p* > 0.001) for overall PNNs (U = 8,000), PNN+/PV + cells (U = 7,000) and PNN+/PV− cells (U = 4,000), these results shoud be taken into consideration acautiously (Supplementary Figure S1). PNN+/PV− staining intensity was not affected by MD in any of the examind mPFC areas. In the hippocampus, PNN+ intensity was not altered by MD either around PV+ or around PV- neurons (Figures 4D–F).

### Maternal deprivation increased inhibition in the mPFC

**FIGURE 5:**
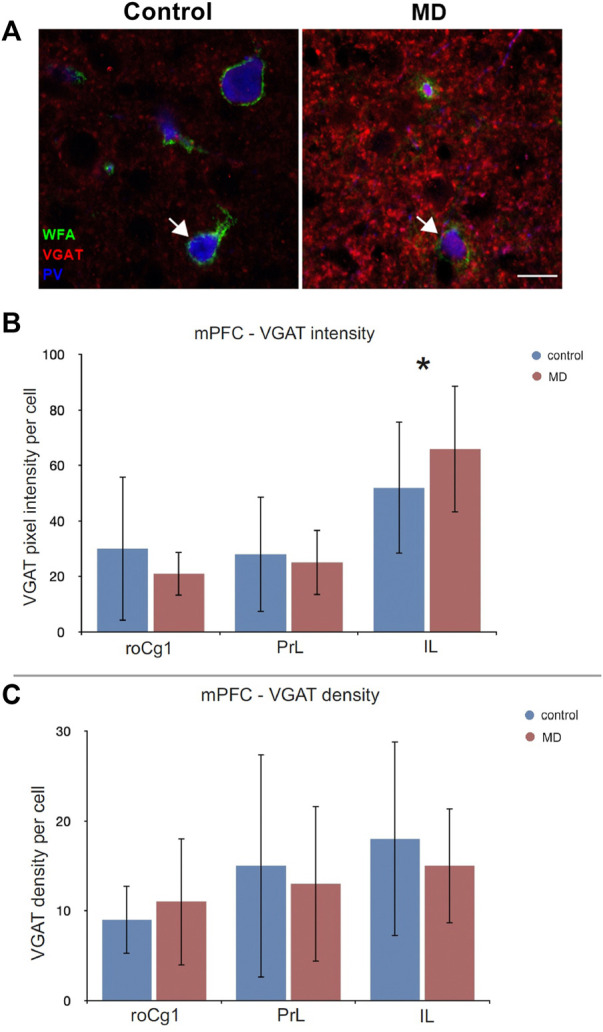
Effects of MD on perisomatic VGAT + puncta intensity and density in the mPFC. **(A)** Images of triple WFA/VGAT/PV immunofluorescence showing VGAT + terminals projecting on the PNN+/PV + cells (arrow) in the subregions of the mPFC. *Bar charts* represent quantifications of the results as means ± SD. MD rats had increased intensity of VGAT + puncta in the IL **(B)**, and no changes in VGAT density in the mPFC **(C)**. Total numbers of evaluated cells per animal: roCg1 = 48, PrL = 69, IL = 56. *p < 0.001. Rostral part of area one of cingulate cortex (roCg1), infralimbic cortex (IL), maternally deprived rats (MD), prelimbic cortex (PrL). Scale bar in A = 15 μm.

To establish whether the changes in the perineuronal nets affected synaptic terminals, we examined perisommatic inhibitory (VGAT+) puncta around pyramidal cells. The analysis of PNN+/PV+ cells for density and signal intensity of perisomatic VGAT puncta revealed VGAT puncta intensity significantly increased only in IL of MD animals (GEE, control = 18 ± 10.86 vs. MD = 15 ± 6.40, *n* = 5, *p* < 0.001, Figures 5A,B). There were no significant difference between density and intensity of MD and control rats in other mPFC regions (Figures 5B,C).

### Maternal deprivation affected the volume of mPFC, without altering the hippocampus

**FIGURE 6:**
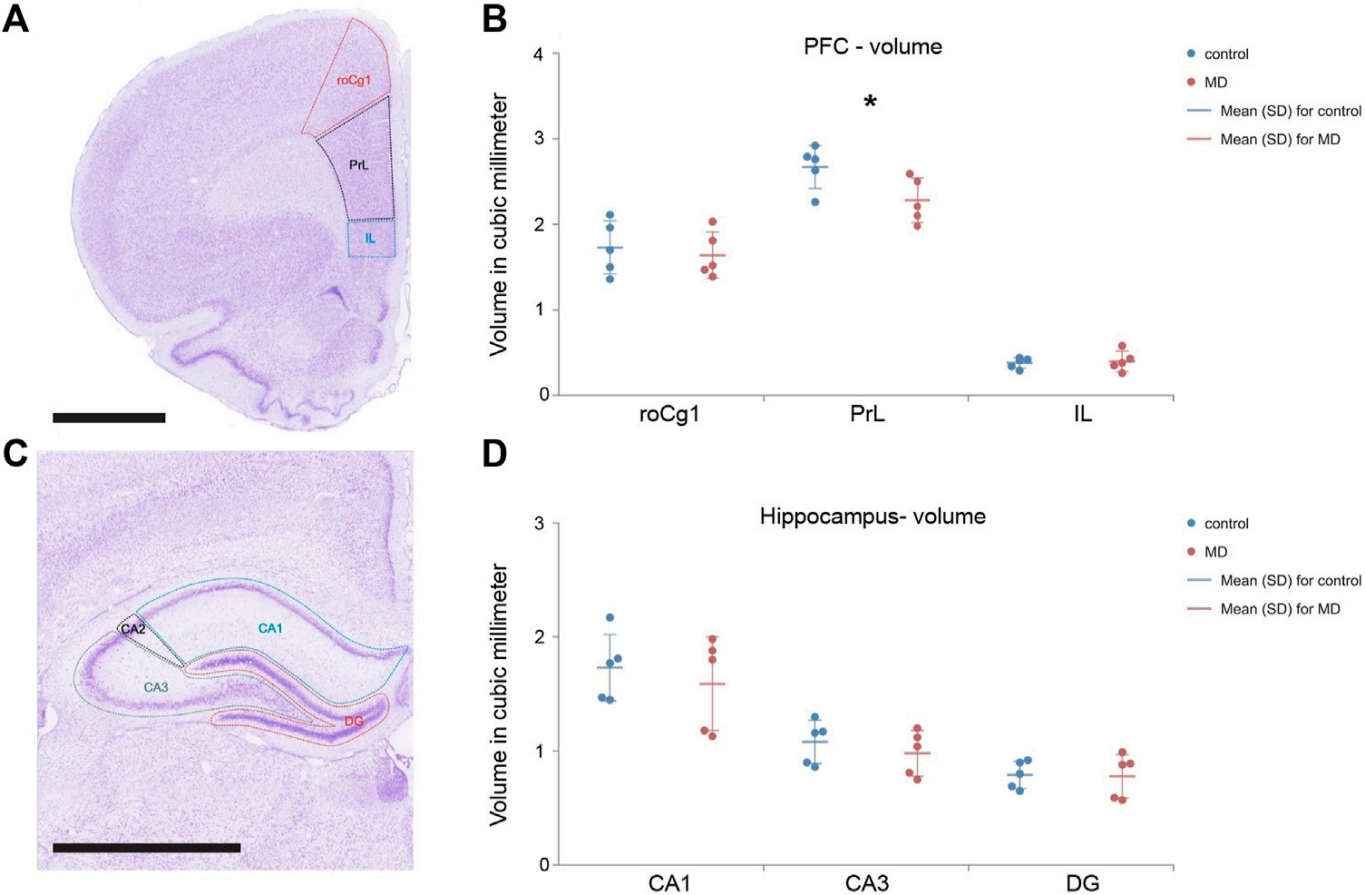
Total volume of the mPFC and hippocampus divided into subfields. Representative micrograph of the cresyl violet stained section of the mPFC divided into subfields roCg1, PrL and IL **(A)** and hippocampus divided into subfields CA1, CA3 and DG **(C)** with delineated regions for volume analysis. *Dot plot graphs* represent s quantification of the results as means ± SD (horizontal bars) and observed values (dots) in the respective the mPFC **(B)** and hippocampus **(D)**. MD rats had decreased volume of the PrL comparing to Control, *p < 0.001. Hippocampal subfields CA1 (CA1), CA2 (CA2), and CA3 (CA3), rostral part of area 1 of cingulate cortex (roCg1), dentate gyrus (DG), infralimbic cortex (IL), prelimbic cortex (PrL). Scale bar = 2 mm.

Volume of the mPFC after MD was changed in area-specific manner. Only the PrL volume was significantly reduced in MD (GLM, control = 2.67 ± 0.25 mm^3^ vs. MD = 2.28 ± 0.26 mm^3^, *n* = 5, *p* < 0.001, Figures 6A,B) while volumes of the roCg1 and IL were not affected (Figure 6B). We found no changes in the volume of hippocampal subregions DG, CA3, CA1 in MD rats comparing to control (Figures 6C,D).

## Discussion

Our findings indicate that MD affected PNNs density and intensity, expression of VGAT puncta and volume of the mPFC in an area specific manner, without causing changes in the hippocampus. The analysis of the mPFC subregions revealed that neonatal stress reduced the density of overall PNNs and PNNs surrounding PV+ cells in the roCg1. Maternal deprivation reduced the intensity of overall PNNs and PNN+/PV+ cells in the PrL, and lead to decrease in both density and intensity of PNNs and PNN+/PV+ cells in the IL. No changes were detected in PNN+/PV− cells in any of the mPFC areas. Analysis of VGAT inhibitory puncta projecting on PNN+/PV+ cells, showed increase in the intensity in the IL after MD. Finally, volume of the PrL was reduced upon MD protocol, where no other regions of the mPFC or the hippocampus were affected.

Results from our study showed that MD decreased overall density of PNNs in the IL, as well as in the roCg1, which is in line with the study of Gildawie et al. (2020), where both males and females were reported to have overall reduction of PNN density in the IL after 4 h/day of maternal deprivation from PND2-20. Contrary to our results, where no changes in PNN density were found in the Prl, aforementioned study showed decreased PNN density in the PrL in both male and female rats exposed to MD (Gildawie et al., 2020). Also, in Gildawie et al., 2020, PNN+/PV− density was found decreased in the IL of adult female rats, while our results showed density reduction in PNNs surrounding PV+ neurons in the IL and roCg1 of young adult males. In addition, by using a different MD protocol, a recent study showed that maternal separation of 3 h/day from PND2-14, had no impact on the number of PV+ cells and PNNs numbers in the PrL and IL (Richardson et al., 2021).

In the PrL and IL, our results showed a decrease in perineuronal nets intensity around PV+ and PV− neurons, following MD. These changes are in good agreement with the work of Ueno et al., 2018, where 50 days of physical and social stress following weaning, decreased WFA fluorescence intensity in the PrL and IL. Postnatal development of the prefrontal cortex continues throughout neonatal age, juvenile age and adolescence, characterized by higher magnitude of synaptogenesis and synaptic pruning comparing to other cortical regions (Elston et al., 2009). Because of that, mPFC is especially sensitive to stress, not only in early life (Arnsten, 2009; Teicher et al., 2016) but also in adolescence (Koseki et al., 2009). Thus, when a traumatic event disrupts physiological development of mPFC during weaning and juvenile age, it could lead to similar effects in observed structures, such as PNNs.

The reduction of PNN density in the roCg1 and IL, and reduction of PNN intensity in the PrL and IL, may point to the regional difference in the sensitivity to the MD. Generally, reduction of PNNs either in density or intensity is caused by the increase of proteolytic enzymes that degrade the constituents of PNNs (Stamenkovic et al., 2017). In that manner, complete loss of PNNs, measured through decrease in density, indicates high level of activity of proteolytic enzymes, while reduction in PNN intensity might suggest that proteolytic degradation of PNNs is at lower levels. Experimental protocols from our and the abovementioned studies differed in the type, period, and duration of stress, but having in mind PNNs activity dependent expression, exposure to any stressful conditions could lead to consequential decrease in the WFA staining density and intensity. On the other hand, different maternal deprivation protocols and sex-specific response to stress, might lead to differences in the affected populations of neurons surrounded with PNNs. We speculate that such discrepancies may be caused by different separation protocols employed between the studies (24 h MD at PND9 vs. 4 h/day MD from PND2-20).

By performing the same stress protocol as in our previous studies (Aksic et al., 2021; Poleksic et al., 2021), we confirmed the absence of changes in PV+ cell density after MD in the IL. However, to our surprise no significant changes in the PV interneuron density were detected in the roCg1 and PrL after MD. These differences from our previous findings can be explained in part by using different microscopy and analysis approaches. Specifically, although using the same MD protocol, Poleksic et al., 2021 separated superficial from deep layers, and used a different scanning system (Axio Scan. Z1), and magnification (×20), while in this study deep and superficial layers were analyzed together and images were acquired at confocal laser scanning microscope (LSM 500), with ×40 magnification.

In other studies where no early life stress protocols were conducted, it was discovered that PV staining intensity is dependent of the presence or absence of PNNs, and that it positively correlates with WFA staining intensity in the PrL (Carceller et al., 2020). Additionally, PNN+/PV+ interneurons tend to have more vesicular inhibitory transmitter transporter (VGAT)+ and vesicular glutamate transporter 1 (VGlut1)+ boutons, as well as longer axon initial segments in the PrL, while the number of VGAT+ boutons decreased after enzymatic degradation of PNNs by chondroitinase ABC (Carceller et al., 2020). These results highlight the importance of PNNs in regulation of the synaptic input of PV+ neurons, thus contributing to the synchronization of cortical circuitry in the mPFC. Our results support this view by emphasizing that changes in the PNN+ intensity originate from changes in PNN+ intensity around PV+, and not PV- cells. Therefore, this suggests the importance of both PV+ cells and PNNs in regulation of neuronal activity in the mPFC following early life trauma. Given that the main role in regulation of neuroendocrine stress in the mPFC plays its infralimbic part (Radley et al., 2006) we emphasize the importance of PNN+/PV+ cells in the IL, whereby compared to other regions of the mPFC examined in this study, the IL showed most prominent and consistent changes following MD. Medial prefrontal cortex plays a pivotal role in the regulation of tone fear extinction and cognitive flexibility (Nett and LaLumiere, 2021), functions previously reported to be affected by early life stress (Baudin et al., 2012; Stamatakis et al., 2016). Interestingly, reversal learning and attention set shift, two main aspects of cognitive flexibility were not altered by enzymatic degradation of PNNs in the mPFC of adult rats (Paylor et al., 2018). The absence of cognitive inflexibility may be explained by the maturation of the PFC circuits in the adult rats. However, in our study, the stressful event occurred during early postnatal period, when the intensely developing mPFC is more vulnerable to the effects of early life experiences (Kolb et al., 2012). Changes in the PNNs reflected on the inhibition balance in the mPFC, where we found significant increase of VGAT+ puncta projecting on the PNN+/PV+ cells in the IL of MD rats. Investigation of the effect of chronic stress on the IL subregion of the mPFC was conducted by McKlveen et al., 2016, using patch clamp on the IL pyramidal neurons. They showed that 14 days of chronic variable stress in adulthood leads to increase in perisomatic GABA release (McKlveen et al., 2016). Nonetheless, the increase in density of VGAT+ puncta was found in the IL of male and female mice after conducting 15 days of peripuberty stress (Bueno-Fernandez et al., 2021). Next, Lensjø et al., 2017 showed the increase of postsynaptic inhibitory puncta after enzymatic removal of PNNs in the primary visual cortex. Although the described stress protocols differ from the early life stress model, the increase in inhibitory puncta could suggest a general mechanism of hypoactivity of the mPFC, and other brain areas, as a response to stressful experiences and PNN removal.

In the study where no stress protocols were conducted, but solely the application of chondroitinase ABC that degrades PNNs in the mPFC did not lead to the changes in the VGAT perisomatic puncta on PNN+/PV+ cells (Carceller et al., 2020). Contrary to that, our experiment of 24 h MD, showed reduced intensity and density of PNNs, but increased VGAT puncta projected on the PNN+/PV+ cells in the IL of mPFC. Such findings point that in conditions of maternal deprivation, if PNNs are degraded and no longer properly controlling and stabilizing inhibitory projections on the PV+ cells, increase in inhibition could possibly lead to the shift in the excitation/inhibition balance.

Maternal deprivation caused reduction of volume in the PrL, which is in line with findings of Sarabdjitsingh et al., 2017, where 24 h MD on the PND3 caused volumetric reduction of the mPFC, measured by MRI. Our results for the volume of the hippocampus are in contrast with findings of Aksić et al. (2013), where a reduction of hippocampal volume after 24 h MD on the PND9 was described. Taken together, changes in volume of the PrL upon MD did not alter either density of PNNs or PV+ cells.

Regarding the other neocortical areas, MD did cause reduction in volume of the retrosplenial cortex (Aksić et al., 2013), decrease of glutamic acid decarboxylase in the temporal cortex (Janetsian-Fritz et al., 2018) and changes in purinergic receptors in the insular cortex (Zhang et al., 2018). These findings indicate that observed changes in our study are not limited to the mPFC, but MD is affecting brain regions involved in memory, processing of auditory information and perception of pain.

Our study reported no changes in the density or intensity of the hippocampal PNNs, neither the ones surrounding PV+ nor the ones surrounding PV− neurons, after MD. Likewise, a more severe physical and social juvenile stress protocol, conducted by Ueno et al., 2018, did not cause any changes in the PNN density or percentage of PNNs around PV+ neurons in any of the hippocampus subregions. The only difference found was a decreased WFA fluorescence intensity, solely in the CA1 region (Ueno et al., 2018). However, chronic MD demonstrated an increase in WFA intensity in the DG of ventral hippocampus, while density of PNNs was not affected (Murthy et al., 2019). Given the fact that functions of the dorsal and ventral hippocampus differ (Fanselow and Dong, 2010), it is not surprising that MD does not affect both regions in the same manner. As previously discussed, some discrepancies in the results can also be explained by differences in stress protocols.

In our study, the medial prefrontal cortex showed an increased sensitivity to early life stress. Although different models of stress at early age may lead to slightly non-consistent results, it is clearly shown that the development of PNNs associated with PV + interneurons in the mPFC is affected by neonatal and juvenile stress. Consequently, these developmental changes could have long lasting effects on the inhibitory circuitry, resulting in a disturbed excitatory/inhibitory (E/I) balance (McKlveen et al., 2019). In this regard, Spijker et al., 2020 proposed a model of ECM changes following exposure to stressful events in early life. There, physiological expression of PNNs and PV that ensures optimal synaptic connectivity, once disturbed by acute stress, leads to increased connectivity, possibly by decreasing expression of PNNs (Spijker et al., 2020). Decreased intensity of PNNs in the mPFC, found in our study, together with increased inhibition could reflect the acute phase of the stress response where synapses are not being stabilized by PNNs. In adulthood, after E/I balance is impaired upon early life stress, more decomposition of ECM molecules could reduce excitatory inputs on PV+ interneurons, thus decreasing overall inhibition in the circuitry (Riga et al., 2017; Spijker et al., 2020). Early life stress, such as maternal deprivation, affects the curve of PNNs development in the mPFC, by prolonging the low PNNs numbers and intensity state into the adulthood, possibly preventing stabilization of projected synapses on the PV+ interneurons (Sultana et al., 2021). This view is also supported by our recent findings of increased plasticity/stability ratio, indicated by higher BNDF expression in the PFC of neonatal and young adult MD rats (Poleksic et al., 2021).

The limitation of your study is that we used only male rats, due to the limited capacities and resources. Thus, further study of PNNs and PV expression in female and male rats, together with measurements of inhibition and excitation in the mPFC after 24 h MD protocol is warranted.

## Conclusion

The results of our study show that MD negatively affects PNNs and inhibitory projections around PV+ interneurons in the medial prefrontal cortex, causing area specific reduction that are most prominent in the infralimbic cortex. These findings highlight the importance of PNNs as regulators of PV+ interneuron function during brain development, showing for the first time the changes in the inhibition together with altered PNNs around PV + cells in this MD model. Future studies focusing on excitatory synaptic plasticity and connectivity should contribute to better understanding of how early life stress shapes the development of PNNs.

## Data Availability

The raw data supporting the conclusion of this article will be made available by the authors, without undue reservation.
