# Supplementary material for: The impact of early life maternal deprivation on the perineuronal nets in the prefrontal cortex and hippocampus of young adult rats
Source: Front Cell Dev Biol. 2022 Nov 28;10:982663. doi: 10.3389/fcell.2022.982663 (PMC9742529; doi:10.3389/fcell.2022.982663)
Supplement: Supplementary file 1 [file Table1.docx]

**Supplementary Table 1. Number of investigated PNNs and PV cells in the medial prefrontal cortex (mPFC)**

|  | | | | | |
| --- | --- | --- | --- | --- | --- |
| **Animal** | **Number of investigated PV+ cells** | **Number of investigated PNNs** | **Number of investigated PNN+/PV+ cells** | **Number of investigated PNN+/PV- cells** | **Number of investigated PNNs with VGAT puncta** |
| *mPFC-roCg1* | | | | | |
| Control 1 | 120 | 56 | 52 | 4 | 7 |
| Control 2 | 59 | 46 | 31 | 15 | 3 |
| Control 3 | 117 | 58 | 53 | 5 | 6 |
| Control 4 | 13 | 78 | 7 | 71 | 3 |
| Control 5 | 132 | 84 | 82 | 2 | 7 |
| Maternal deprivation 1 | 93 | 63 | 59 | 4 | 6 |
| Maternal deprivation 2 | 77 | 45 | 38 | 7 | 6 |
| Maternal deprivation 3 | 84 | 46 | 39 | 7 | 4 |
| Maternal deprivation 4 | 81 | 44 | 38 | 6 | 6 |
| Maternal deprivation 5 | 65 | 29 | 21 | 8 | 0 |
| *mPFC-PrL* | | | | | |
| Control 1 | 173 | 129 | 109 | 20 | 8 |
| Control 2 | 122 | 100 | 75 | 25 | 4 |
| Control 3 | 130 | 74 | 58 | 16 | 7 |
| Control 4 | 121 | 91 | 56 | 45 | 7 |
| Control 5 | 95 | 74 | 69 | 5 | 6 |
| Maternal deprivation 1 | 115 | 108 | 81 | 27 | 7 |
| Maternal deprivation 2 | 93 | 62 | 53 | 9 | 7 |
| Maternal deprivation 3 | 142 | 92 | 76 | 16 | 8 |
| Maternal deprivation 4 | 107 | 67 | 59 | 8 | 8 |
| Maternal deprivation 5 | 129 | 97 | 78 | 19 | 7 |
|  |  |  |  |  |  |
| *mPFC-IL* | | | | | |
| Control 1 | 38 | 24 | 22 | 2 | 8 |
| Control 2 | 46 | 37 | 31 | 6 | 8 |
| Control 3 | 23 | 18 | 14 | 4 | 4 |
| Control 4 | 48 | 30 | 28 | 2 | 5 |
| Control 5 | 50 | 34 | 32 | 2 | 8 |
| Maternal deprivation 1 | 36 | 26 | 22 | 4 | 8 |
| Maternal deprivation 2 | 30 | 16 | 12 | 4 | 7 |
| Maternal deprivation 3 | 48 | 24 | 23 | 1 | 0 |
| Maternal deprivation 4 | 37 | 17 | 13 | 4 | 4 |
| Maternal deprivation 5 | 36 | 16 | 9 | 7 | 4 |
