# Supplementary material for: The impact of early life maternal deprivation on the perineuronal nets in the prefrontal cortex and hippocampus of young adult rats
Source: Front Cell Dev Biol. 2022 Nov 28;10:982663. doi: 10.3389/fcell.2022.982663 (PMC9742529; doi:10.3389/fcell.2022.982663)
Supplement: Supplementary file 2 [file Table2.docx]

**Supplementary Table 2. Number of investigated PNNs and PV cells in the hippocampus**

|  | | | | |
| --- | --- | --- | --- | --- |
| **Animal** | **Number of investigated PV+ cells** | **Number of investigated PNNs** | **Number of investigated PNN+/PV+ cells** | **Number of investigated PNN+/PV- cells** |
| *Hippocampus-CA1* | | | | |
| Control 1 | 50 | 35 | 21 | 6 |
| Control 2 | 35 | 35 | 26 | 9 |
| Control 3 | 62 | 44 | 28 | 16 |
| Control 4 | 44 | 24 | 22 | 4 |
| Control 5 | 44 | 40 | 24 | 16 |
| Maternal deprivation 1 | 27 | 24 | 21 | 3 |
| Maternal deprivation 2 | 62 | 38 | 31 | 7 |
| Maternal deprivation 3 | 44 | 28 | 17 | 9 |
| Maternal deprivation 4 | 39 | 21 | 14 | 7 |
| Maternal deprivation 5 | 70 | 73 | 24 | 49 |
| *HIppocampus-CA3* | | | | |
| Control 1 | 31 | 34 | 12 | 22 |
| Control 2 | 43 | 59 | 19 | 40 |
| Control 3 | 54 | 60 | 23 | 37 |
| Control 4 | 28 | 20 | 10 | 10 |
| Control 5 | 25 | 59 | 22 | 37 |
| Maternal deprivation 1 | 27 | 46 | 14 | 32 |
| Maternal deprivation 2 | 50 | 56 | 24 | 32 |
| Maternal deprivation 3 | 30 | 25 | 13 | 12 |
| Maternal deprivation 4 | 33 | 28 | 16 | 12 |
| Maternal deprivation 5 | 81 | 31 | 19 | 12 |
| *Hippocampus-DG* | | | | |
| Control 1 | 29 | 17 | 9 | 8 |
| Control 2 | 21 | 37 | 14 | 23 |
| Control 3 | 55 | 23 | 20 | 3 |
| Control 4 | 27 | 13 | 8 | 5 |
| Control 5 | 55 | 24 | 22 | 2 |
| Maternal deprivation 1 | 18 | 10 | 7 | 3 |
| Maternal deprivation 2 | 58 | 28 | 24 | 4 |
| Maternal deprivation 3 | 46 | 10 | 9 | 1 |
| Maternal deprivation 4 | 36 | 16 | 12 | 4 |
| Maternal deprivation 5 | 81 | 31 | 19 | 12 |
