# Supplementary material for: The impact of early life maternal deprivation on the perineuronal nets in the prefrontal cortex and hippocampus of young adult rats
Source: Front Cell Dev Biol. 2022 Nov 28;10:982663. doi: 10.3389/fcell.2022.982663 (PMC9742529; doi:10.3389/fcell.2022.982663)
Supplement: Supplementary file 3 [file Table3.docx]

| ***mPFC*** | | | | | | |
| --- | --- | --- | --- | --- | --- | --- |
| **Measured parameters** | ***roCg1*** | | ***PrL*** | | ***IL*** | |
|  | *W*_1,9_  *value* | *p value* | *W*_1,9_ *value*  *U value* | *p value*  *p value* | *W*_1,9_ *value* | *p value* |
| PV+ density |  |  |  |  |  |  |
| Overall PNN density | 18.904 | <0.001 |  |  | 17.820 | <0.001 |
| PNN+/PV+ density | 12.373 | <0.001 |  |  | 15.965 | 0.001 |
| PNN+/PV- density |  |  |  |  |  |  |
| Overall PNN intensity | 2,175 | 0,140 | 23.280  8.000 | <0.001  >0,001 | 14.552 | <0.001 |
| PNN+/PV+ intensity | 1,880 | 0,170 | 26.872  7.000 | <0.001  >0,001 | 16.891 | <0.001 |
| PNN+/PV- intensity | 4,582 | 0,032 | 6,151 | 0,013 | 0,014 | 0,906 |
| VGAT puncta density | 0,851 | 0,356 | 1,876 | 0,171 | 2,406 | 0,121 |
| VGAT puncta intensity | 4,894 | 0,027 | 0,062 | 0,804 | 46.650 | <0,001 |
| Volume | 1,805 | 0,179 | 17.246 | <0,001 | 2,293 | 0,130 |
| ***Hippocampus*** | | | | | | |
| **Investigated parameters** | ***CA1*** | | ***CA3*** | | ***DG*** | |
|  | *W*_1,9_  *value* | *p value* | *W*_1,9_  *value* | *p value* | *W*_1,9_  *value* | *p value* |
| PV+ density | 0,011 | 0,915 | 5,811 | 0,016 | 1,352 | 0,245 |
| Overall PNN density | 1,163 | 0,281 | 1,410 | 0,235 | 2,620 | 0,106 |
| PNN+/PV+ density | 1,050 | 0,305 | 0,035 | 0,853 | 2,694 | 0,101 |
| PNN+/PV- density | 6,737 | 0,009 | 1,770 | 0,183 | 0,778 | 0,378 |
| Overall PNN intensity | 0,524 | 0,469 | 0,918 | 0,338 | 7,564 | 0,006 |
| PNN+/PV+ intensity | 0,009 | 0,926 | 1,141 | 0,285 | 3,027 | 0,082 |
| PNN+/PV- intensity | 2,784 | 0,095 | 1,596 | 0,206 | 4,545 | 0,033 |
| VGAT puncta density | / | / | / | / | / | / |
| VGAT puncta intensity | / | / | / | / | / | / |
| Volume | 0,227 | 0,634 | 0,636 | 0,425 | 0,012 | 0,913 |

**Supplementary Table 3. Treatment effect results for each parameter in investigated brain regions. GLM was performed for all parameters except for VGAT measurements (GEE test). As in PrL region Grubb’s test confirmed outliers, additional non-parametric Mann-Withney test was performed.**
