# Supplementary material for: The impact of early life maternal deprivation on the perineuronal nets in the prefrontal cortex and hippocampus of young adult rats
Source: Front Cell Dev Biol. 2022 Nov 28;10:982663. doi: 10.3389/fcell.2022.982663 (PMC9742529; doi:10.3389/fcell.2022.982663)
Supplement: Supplementary file 4 [file Image1.pdf]

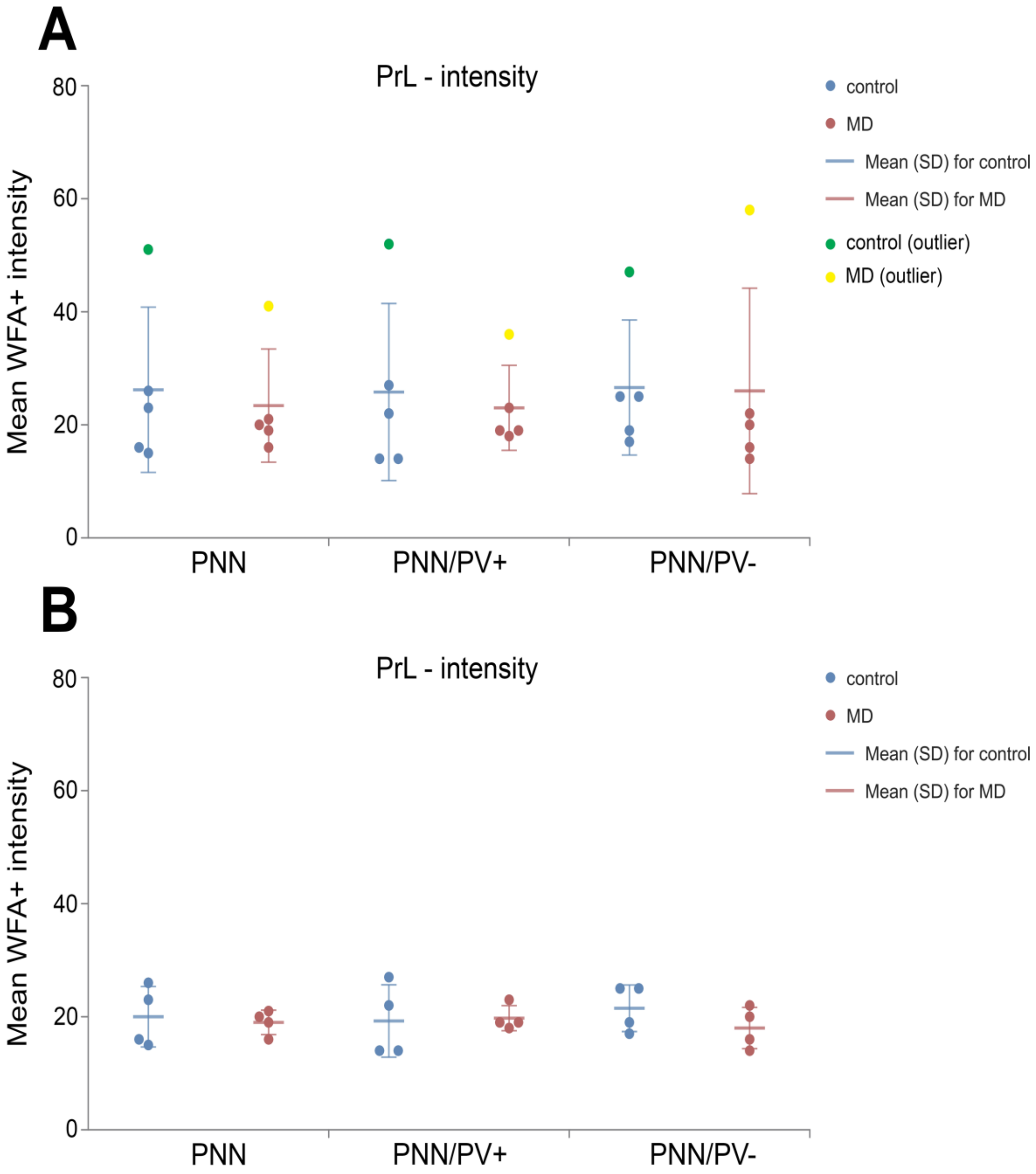

Supplementary figure 1. Effects of MD on WFA+ perineuronal nets intensity in the PrL. *Dot plot graphs* representing quantifications of the results as means ± SD (horizontal bars) and observed values (dots). **(A)** Grubb's test confirmed outliers that are marked with green (control) and yellow (MD). **(B)** No significant difference was observed after excluding marked outliers. Maternally deprived rats (MD), prelimbic cortex (PrL).
